# The role of ECL2 in CGRP receptor activation: a combined modelling and experimental approach

**DOI:** 10.1098/rsif.2013.0589

**Published:** 2013-11-06

**Authors:** Michael. J. Woolley, Harriet A. Watkins, Bruck Taddese, Z. Gamze Karakullukcu, James Barwell, Kevin J. Smith, Debbie L. Hay, David R. Poyner, Christopher A. Reynolds, Alex C. Conner

**Affiliations:** 1 Warwick Medical School, University of Warwick, Coventry CV4 7AL, UK; 2 School of Biological Sciences, University of Auckland, 3 Symonds Street, Private Bag 92 019, New Zealand; 3 School of Biological Sciences, University of Essex, Wivenhoe Park, Colchester CO4 3SQ, UK; 4 School of Life and Health Sciences, Aston University, Aston Triangle, Birmingham B4 7ET, UK

**Keywords:** calcitonin gene-related peptide, class B G-protein-coupled receptor, loop modelling, site-directed mutagenesis, calcitonin receptor-like receptor

## Abstract

The calcitonin gene-related peptide (CGRP) receptor is a complex of a calcitonin receptor-like receptor (CLR), which is a family B G-protein-coupled receptor (GPCR) and receptor activity modifying protein 1. The role of the second extracellular loop (ECL2) of CLR in binding CGRP and coupling to Gs was investigated using a combination of mutagenesis and modelling. An alanine scan of residues 271–294 of CLR showed that the ability of CGRP to produce cAMP was impaired by point mutations at 13 residues; most of these also impaired the response to adrenomedullin (AM). These data were used to select probable ECL2-modelled conformations that are involved in agonist binding, allowing the identification of the likely contacts between the peptide and receptor. The implications of the most likely structures for receptor activation are discussed.

## 1. Introduction

The extracellular loops of G protein-coupled receptors (GPCRs) are important for receptor function. They contribute to protein folding, provide structure to the extracellular region and mediate movement of the transmembrane (TM) helices on activation. The second extracellular loop (ECL2) is of significance for ligand binding and receptor activation [1–3]. In family B (or secretin-like) GPCRs, it is the most conserved and often the longest of all the ECLs, and so is in a good position to interact with the endogenous peptide agonists for these receptors and is in a prominent central position to mediate conformational changes [1]. The peptide ligands typically contain 30–50 amino acids. The family B receptors are characterized by a large N-terminal domain (approx. 100 amino acids), which interacts with the C-termini of their cognate peptide ligands [4]. The N-termini of these peptides interact with the ECLs and the TM region of the receptors resulting in activation [4,5]. ECL2 has been implicated in agonist binding at the GLP-1, secretin and CRF1 receptors [6–10]. For the GLP-1 receptor, ECL2 plays an important role in directing coupling towards stimulation of ERK1/2 activation versus Gs and activation of adenylate cyclase [7]. However, the molecular basis for this observation remains obscure.

Several distinct conformations have been recognized for ECL2 in family A GPCRs, ranging from the ‘lid’ seen in rhodopsin, which encloses the bound retinal ligand, to the extended sheet seen for most of the peptide GPCRs [11–13]. Movement of ECL2 seems to be important in the activation of family A GPCRs; this is linked to agonist-induced changes in TM5 and helps propagate these changes to other parts of the GPCR [11,14].

There is currently no crystal structure showing how a peptide agonist binds to the ECLs or TM domain of a family B GPCR. A number of models have been proposed based on cross-linking and mutagenesis data [8,15,16] but it has proved extremely difficult to accurately predict the ECL conformations by this approach or by simulation [17]. Given the variety in family B peptide sequences [5,17], it seems likely that there is no single mode of peptide binding to the ECLs of family B GPCRs [18].

We have previously used a combination of mutagenesis and computation to produce a model indicating how the TM domain of the calcitonin receptor-like receptor (CLR) can interact with Gs [19]. CLR is the GPCR component of the calcitonin gene-related peptide (CGRP) receptor. CGRP is part of a peptide family that also includes adrenomedullin (AM), calcitonin and amylin and is involved in heart disease and migraine [20]. CLR interacts with a single TM protein, receptor activity-modifying protein 1 (RAMP1), in order to bind CGRP and also AM [21], though AM interacts with CLR more strongly in the presence of RAMP2. Using an independent modelling approach, Wootten *et al*. [22] have produced a broadly similar model for the GLP-1 receptor, which indicates how agonists can activate the receptor by interacting with different TM residues. Using both mutagenesis and computation is a powerful strategy for studying the activation of GPCRs [12], especially given that structural techniques for instance crystallography give static pictures of what is fundamentally a dynamic process.

In previous work, we have examined the role of the first and third ECLs (ECL1/3) of the CGRP receptor. However, only a small number of residues within ECLs 1 and 3 were implicated in CGRP binding [18]. Consequently, we have now addressed the role of ECL2 in this receptor. In an extension of the strategy used to examine the TM residues of CLR, we have experimentally identified CLR residues that reside within the last turn of TM4, ECL2 and the first turn of the turn of TM5 and that are key for CGRP and AM interactions with the CGRP receptor. We have then used all of our data for each ECL and the TM domain, in combination with heuristic loop modelling, molecular dynamics, docking and sequence analysis to model the interaction between the N-terminus of the CGRP peptide and CLR, based on a model of the Gs-coupled state of the CGRP receptor.

## 2. Material and methods

### 2.1. Preparation of mutants, transfection, receptor expression and radioligand binding

The preparation of mutants and molecular biology was as described earlier [23]. An HA-tagged CLR construct was used to allow measurement of cell surface receptor expression by ELISA [20,23]. Mutants were transfected into Cos 7 cells using PEI [20,23]. Radioligand binding was carried out on cell membranes using [^125^I] iodohistidyl-human alpha CGRP (Perkin Elmer) [20,23].

### 2.2. Measurement of cAMP

For the investigation of CGRP-mediated activation of the receptor, cAMP was measured using a FRET-based PerkinElmer LANCE cAMP 384 kit according to manufacturer's instructions. Briefly, transfected cells were removed from the plate with trypsin EDTA, washed with phosphate-buffered saline and resuspended in assay stimulation buffer (SB; phosphate-buffered saline + 0.1% (w/v) bovine serum albumin + 0.5 mM isobutylmethylxanthine). Cells were counted with a haemocytometer and the appropriate cell number resuspended in SB + 1/100 AlexaFluor 647-anti cAMP antibody at an assay concentration of 2000 cells/10 µl. A total of 2000 cells/well were loaded onto a 96-well white Optiplate (PerkinElmer) and were concentration-dependently stimulated in triplicate with a logarithmic increase of CGRP diluted in SB from 10^−6^ to 10^−12^ M with SB as a basal point. The plate was incubated in the absence of light for 30 min at room temperature before 20 µl/well of detection mix was added. The plate was incubated in the absence of light for a further 60 min. FRET was recorded by excitation at 320 nm and emission at 665 nm. The experimental pEC_50_ values for wild-type (WT) receptor in the electronic supplementary materials, table S1 show some variation, reflecting differences in coupling efficiencies between cells as the data were collected in excess of a year. Such drift has been observed previously with CGRP receptors [24,25]. To control for this, a paired design-test was used so that each mutant was compared in the same experiment against a corresponding WT control. For the investigation of AM-mediated stimulation of the CGRP receptor, cAMP was measured with alphascreen, as described previously [26]. Data were normalized against the maximum fitted response for CGRP or AM; basal cAMP was taken as the fitted minimum.

### 2.3. Data analysis

Curve fitting was done with GraphPad Prism 5 or 6 (GraphPad Software Inc., San Diego, CA, USA). Both this and statistical analysis were as described previously [20].

### 2.4. CLR models

The starting point for the models indicating the interaction between CGRP and ECL2 was a recent model of the active state [19]. The most appropriate X-ray crystal structure model for a fully active GPCR is that of the β_2_-adrenergic receptor (β_2_-AR) coupled to Gs [27], which is similar to the β_2_-AR nanobody stabilized [28] and the rhodopsin active structures stabilized by a C-terminal peptide from transducin [29,30]; these active structures are characterized by the outward tilt of the intracellular end of TM6 that is necessary for G protein binding. In the absence of G protein or G protein-derived peptides, X-ray crystal structures of agonists bound to GPCRs stabilize substates that are not too different to the inactive form [31], i.e. they lack the outward tilt of TM6; in such agonist-bound structures, the conformation of Y^5.58^ is taken to be indicative of the state because in the inactive form, it usually interacts with L^1.63^ and L^8.50^, whereas in the active substate, it stabilizes the active conformation of R^3.50^. Our active structures are stabilized by the Gs C-terminal peptide (R373-L394), and hence included the outward tilt of TM6 [27]. Nevertheless, the conformation of Y^5.58^ (and the tilt of TM6) was monitored to check that active state character was maintained as fully as possible and that the simulations could be terminated should the active structure begin to acquire inactive character. The underlying alignment was based on a novel approach to the class A–class B alignment, aided by a GCR1/class E alignment that was used as a bridge between the class A and class B sequences. This has been repeated using improved methodology but the alignment remains unaltered [32]. The status of GCR1, GCR2 and other putative plant GPCRs has been questioned [33–35], but GCR1 has all the features expected for a GPCR fold [32], while GCR2 was predicted [36] and later shown to be a lanbiotic cyclase [37]. The simulations that underlie the CLR modelling [19] were extended to 100 ns (see the electronic supplementary material).

To model the peptide binding, disulfide-bonded cyclic CGRP_1–7_ was constructed in Maestro and docked to the active CLR model (after 80 ns, i.e. just before the first signs of the onset of inactive character; [19]) using Glide SP [38,39], as described in the electronic supplementary material. The best-scoring pose was verified by sequence analysis, as described below. The CGRP extension (up to residue 32) was modelled onto CGRP_1–7_ using the NMR solution structure of salmon calcitonin (PDB code 2glh) [40], using the alignment of Watkins *et al*. [5].

In the molecular dynamics simulations, the active structure begins to acquire some inactive character after 80 ns (i.e. F^7.53^ switched towards the inactive conformation; see the electronic supplementary material, figure S1). This is well before it would be possible to fully sample the loop conformations, despite high principle components for ECL2 residues; see the electronic supplementary material, figure S1D. Thus, given that the Modeller scoring functions are approximate, we have taken a heuristic approach to determining the ECL2 conformation. We have therefore generated and refined 100 independent loop conformations; suitable conformations were selected if key loop residues identified by the mutagenesis interacted with CGRP or AM to within a cut-off of 5.5 Å. There are many assumptions inherent in this approach, as discussed below in §3.2.5; this is apparent because it was impossible to generate conformations where each significant ECL2 residue interacted with CGRP. Thus, multiple conformations of ECL1 (residues 202–212), ECL2 (residues 274–293) and ECL3 (residues 353–363) were simultaneously generated in the presence of CGRP_1–32_ using Modeller [41–43]. Each conformation is characterized by its DOPE score; a lower score corresponds to a more likely conformation. AM binding to CLR was similarly modelled by simultaneously mutating CGRP to AM and generating the loop conformations within Modeller.

### 2.5. Sequence analysis

The sequences of CGRP and amylin (which binds to the calcitonin receptor (CTR)) from several species were aligned [5,44]. The sequences of amylin and CGRP and the sequences of CTR and CLR were analysed in parallel to identify mutations that are correlated between the CTR–amylin and CLR–CGRP systems, with a view to identifying contact points.

### 2.6. Residue numbering

For amino acids within the proposed ECLs of CLR, only the residue numbers are shown. For residues that are within the TM helices of CLR, a superscript denotes their position using an adaption of the Ballasteros–Weinstein numbering proposed elsewhere [19]. The peptide residue numbers are given as superscripts.

## 3. Results and discussion

### 3.1. Experimental analysis of the CGRP receptor

In this section, the results of an alanine scan of the CGRP receptor will be presented. The implications of these data will be discussed alongside the modelling in §3.2.

#### 3.1.1. Effects of alanine substitution on CGRP-mediated cAMP production

**Figure 1.**
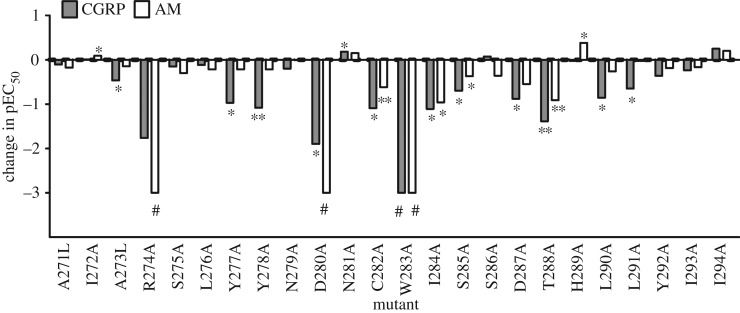
Effect of mutations on the pEC_50_ for AM (white bars) and CGRP (grey bars) at cAMP production. The difference in mean pEC_50_ between the mutant and WT receptor is shown, hence a negative value shows a decrease in potency. Where there was no detectable stimulation of the mutant by peptide (#), an arbitrary value of –3 has been shown in the figure. Data are shown in the electronic supplementary material, table S1. *p < 0.05, **p < 0.01, pEC_50_ of mutant significantly different from that of WT by paired Student's *t*-test or repeated measures ANOVA followed by Dunnett's test as appropriate.

**Figure 2.**
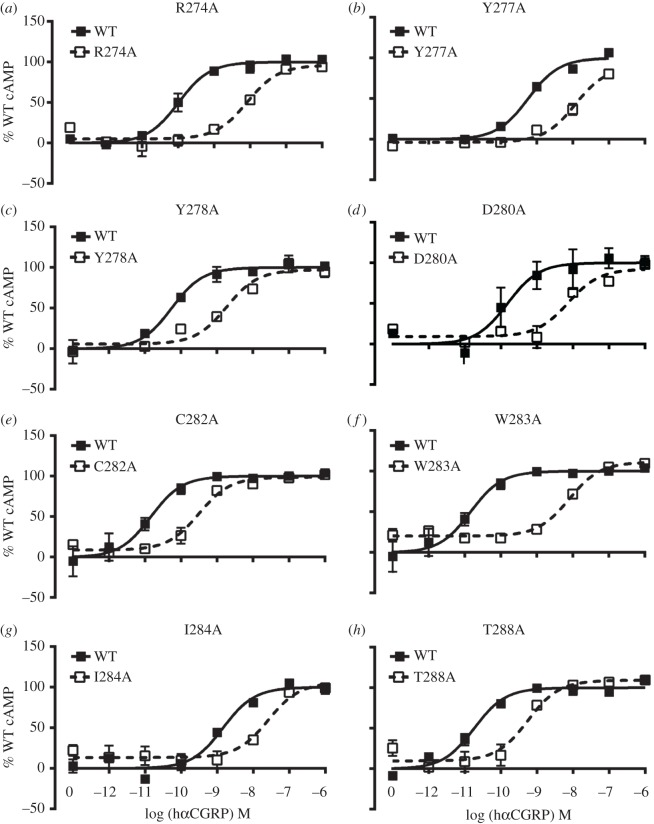
(*a*–*h*) Concentration–response curves of mutants showing changes in pEC_50_ in response to CGRP. Representative curves are shown from experiments repeated at least three times. The curves are normalized to the fitted *E*_max_ for CGRP on the WT receptor, which is defined as 100%.

Twenty-four individual residues of CLR ECL2 from A271 to I294 were mutated to alanine (alanine residues were mutated to leucine) and their ability to respond to CGRP and stimulate cAMP production was investigated when co-expressed with human RAMP1. These residues were selected as they are most likely to incorporate the whole of ECL2 and approximately one turn of helices 4 and 5 immediately adjacent to the loop, based on our previous analysis [19]. Figures 1 and 2 and the electronic supplementary materials, table S1 and figure S2 show that the pEC_50_ values of 14 of the 24 mutants were significantly different to WT, indicating that ECL2 is particularly important for CGRP receptor function. Seven mutants resulted in significant cAMP reduction of more than 10-fold. These are R274A, Y278A, D280A, C282A, W283A, I284A and T288A. Of the remaining seven mutations, N281A showed a small increase in potency and six mutants had small but significant reductions in pEC_50_. Y292A and I294A showed significant but small increases in maximal cAMP response (*E*_max_) (see the electronic supplementary material, table S2).

#### 3.1.2. Cell surface expression of receptors and CGRP binding

Owing to the likelihood of attenuated binding of CGRP, radioligand binding could not be reliably used to provide an estimate of receptor expression for these constructs. Accordingly, an ELISA to measure receptors at the cell surface was used (see the electronic supplementary materials, table S3). All of the mutants were expressed at the cell surface to within approximately 60% of WT levels. Reductions to this level of expression has been previously shown to have little effect on the potency of CGRP-mediated cAMP signalling for this or CTR expressed in Cos 7 cells [45,46] and in this study, there was only a very low correlation between expression and pEC_50_ (*r*^2^ = 0.23). Mutated receptors that had large impairments of cAMP production were examined for their ability to bind CGRP using a radioligand-binding assay (see the electronic supplementary materials, table S4). All of the mutants examined, except Y277A had reduced affinity for the radioligand.

#### 3.1.3. Effects of alanine substitution on AM-mediated cAMP production

**Figure 3.**
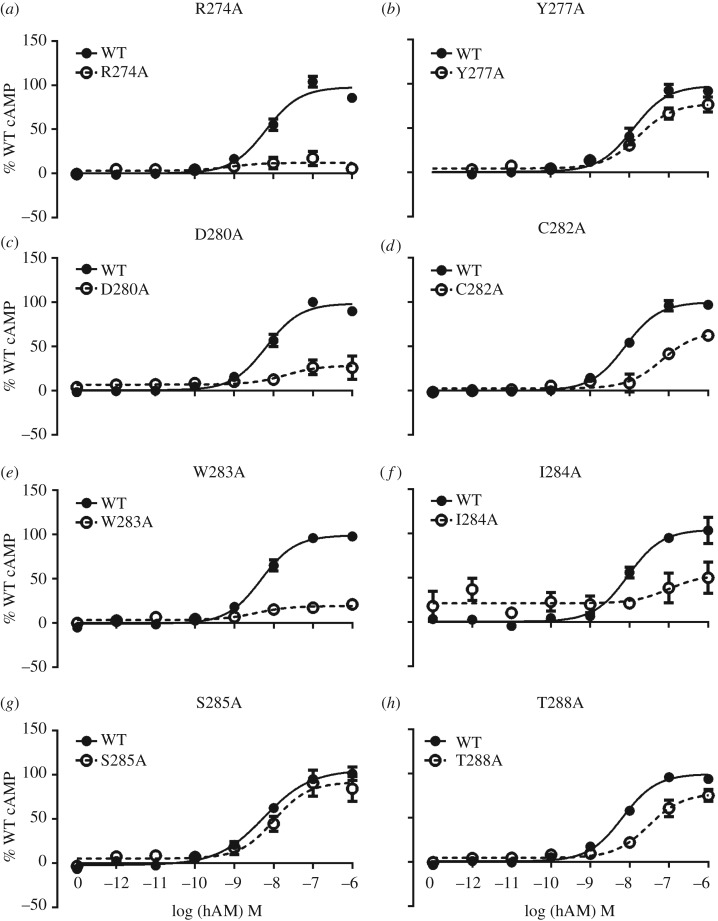
(*a*–*h*) Concentration–response curves of mutants showing changes in cAMP in response to AM. Data are mean ± s.e.m. of combined normalized data from at least three independent experiments. The curves are normalized to the fitted *E*_max_ for AM on the WT receptor, which is defined as 100%.

The CGRP receptor also acts as a functional AM receptor with an affinity of approximately 10-folds less than that for CGRP [47]. To explore the effects of the above mutants further, their ability to respond to AM through cAMP production was subsequently investigated. The majority of the effects were in line with those seen with CGRP (figure 1 and the electronic supplementary material, table S2), the most notable differences being the lack of effects of AM at Y277 and Y278 and for some mutants, it was impossible to measure any activation of the receptor (figure 3). In the case of R274A, W283A and D280A, the response was so low that an *E*_max_ could not be reliably determined. There was especially good agreement between the two agonists for the central area of functional importance ranging from D280 to T288. As with CGRP, many of these alanine substitutions reduced AM potency. The effect of mutations at the N- and C-termini of ECL2 was more variable between the two peptides. At the N-terminus, the CGRP-affecting mutants Y277A or Y278A did not show AM-mediated effects on pEC_50_. However, there were approximately 25% reductions in the maximal cAMP response. At the C-terminus of ECL2, the two leucine mutants (L290A and L291A), which showed a reduction in potency with CGRP, were not significantly affected when stimulated with AM.

#### 3.1.4. Basal activity

In the investigation of CGRP (with Cos 7 cells from a UK source and measuring cAMP with a LANCE assay), N281A and I294A showed a significant increase in basal cAMP signalling (i.e. constitutive ligand-independent signalling activity; see the electronic supplementary material, table S2). These values increased by 9.9 ± 0.9% and 21.8 ± 2.9% above WT, respectively. When the cells were investigated for AM responsiveness (using Cos 7 cells from a New Zealand source and Alphascreen), small but statistically significant elevations were noted for A273L, Y277A and I284A but not N281A and I294A. Thus, the increase in basal cAMP depended on experimental conditions and was always small. In the course of analysing over 200 mutants of CLR [19,20,48], we have observed very few that showed elevated activity, possibly indicating that there are multiple locks in place to keep the receptor in an inactive form; it is possible that the RAMPs might contribute to this.

#### 3.1.5. The importance of the ECL2–TM3 disulfide linkage

As C282 is predicted to form a disulfide bond with C212^3.25^ [19], further mutagenesis was used to explore this, using CGRP as the agonist. The mutant C212A impaired cAMP production in much the same way as C282A (pEC_50_ values: WT 9.62 ± 0.76, C212A 8.10 ± 0.43, *n* = 3, *p* < 0.05, Student's *t*-test); however, the double mutant C212AC282A showed a WT response (pEC_50_ values: WT 9.49 ± 0.11 *n* = 3, C212AC282A 9.41 ± 0.09, *n* = 3), thus implying that the disulfide bond itself is not crucial for CGRP binding or signalling.

#### 3.1.6. The importance of the ECL2/TM5 junction

As noted above, L290A and L291A both showed small but significant reductions in CGRP potency. As movements at the top of TM5 have been implicated in the early stages of activation of the β_2_-AR and rhodopsin [11], the role of residues in this region was probed by further mutagenesis. The individual reduction in EC_50_ values for L290A (approx. eightfold) and L291A (approx. fourfold) was highly exacerbated by an L290AL291A double mutant that reduced the EC_50_ by approximately 500-fold compared with WT (WT pEC_50_ 10.6 ± 0.11, L290AL291A pEC_50_ 7.93 ± 0.13, *n* = 3, *p* < 0.01, Student's *t*-test).

### 3.2. Modelling of ECL2 and interactions with CGRP and AM

#### 3.2.1. Analysis of the Modeller-generated ECL2 conformations

**Figure 4.**
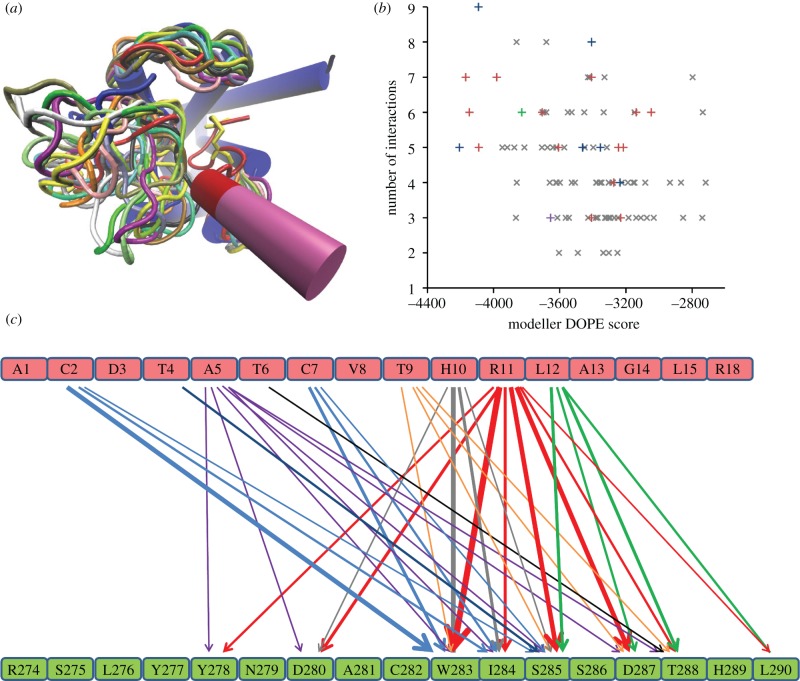
(*a*) A sample (20/100) of ECL1, ECL2 and ECL3 conformations generated by Modeller for the CLR : CGRP complex; each of the 20 loops is shown in a different colour. CLR is shown in blue, helices are shown as cylinders, CGCR_1–12_ is shown in red and CGRP_12–18_, which has relatively few interactions to ECL2, is shown in mauve. The C^2^–C^7^ disulfide bond is shown in yellow. (*b*) A plot of the number of residue–residue interactions between ECL2 and CGRP against the Modeller DOPE score for the CLR conformation; the lower the score, the more probable the loop conformation. Conformations that interact with T^6^ via T288, D287, D280 or both D287 and T288 are shown as red, blue green and purple crosses (+), respectively. Conformation were these residues do not interact with T^6^ are shown as grey crosses (×). (*c*) The interactions between ECL2 residues and CGRP residues as observed over the full set of 100 ECL2 conformations; the thickness of the line is broadly in line with the frequency of the interactions. For clarity interactions observed in fewer than 10 structures are omitted (but see the electronic supplementary materials, figure S3 for the full set of interactions). The coloured lines indicate interactions of specific CGRP residues: D^3^ (brown), A^5^ (purple), T^6^ (black), T^9^ (orange), H^10^ (grey), R^11^, R^18^ (red) and L^12^, L^15^ (green).

The loop conformations spanned a large proportion of the space available to ECL2 (figure 4*a*). The analysis of the full set of interactions for all loop conformations is given in figure 4*b*,*c*. Although this analysis includes high-energy conformations, it is interesting as it highlights general interaction preferences of several amino acids in CGRP and ECL2. ECL2 forms an extended loop; this conformation is often seen as two antiparallel beta-strands in all peptide GPCRs for which structures are available, creating a large interface for peptide–receptor contacts [12]. Here, we re-interpret several previously reported experimental observations that were made in the absence of a modelled structure [44] and that are consistent with our models. D^3^ of CGRP in our model is not in a constrained pocket, hence it makes few interactions with ECL2; indeed this position can accommodate a photoaffinity probe [44]. The fact that it can be readily replaced by arginine in the AM model provides further justification for our model. By contrast, A^5^ of CGRP is sterically constrained and indeed makes a relatively large number of interactions (figure 4*c*), showing that it has many close neighbours. T^6^ is discussed in more detail below; here, the preferred interactions are to T288. In several loop conformations, T^9^ of CGRP interacts with polar residues such as D280, D287 and T288. There is some indirect evidence that H^10^ may also be part of this network that responds to negative charges [44]. L^15^ of CGRP can be replaced by a large benzoyl-phenylalanine moiety with only small changes in affinity (it makes few interactions to ECL2); however, replacement of L^12^ of CGRP causes around a 10-fold decrease (it makes more interactions with ECL2) [44]. Replacement of R^18^ of CGRP by alanine has virtually no effect, and indeed it makes few interactions, but the double alanine mutant R^11^AR^18^A shows 100-fold reduction in affinity [44]. Replacing either of these arginine residues with glutamate caused over a 10-fold reduction in affinity; but replacement with glutamine led to retention of high affinity binding [24,25,49], presumably because glutamine can still donate hydrogen bonds. Indeed, we see that both D280 and D287 are able to interact with R^11^, but as D287 is less significant for AM (where K substitutes for R^11^), it is more likely that D280 is the preferred partner to R^11^, even though D287 makes more interactions. CGRP residues 1, 4, 8, 13 and beyond are predicted to make few, if any contacts with ECL2; this is consistent with the known structure activity relationships for CGRP where they seem to be of only minor importance [44]; this observation justifies the orientation of the helical extension to CGRP_1–7_.

W283 and I284, which reside in the centre of ECL2, make the most interactions; these are either to the region around R^11^ or to the N-terminal region (see below).

Having analysed the pattern of interactions over all loop conformations, we see that this pattern is consistent with known experimental data on the CGRP peptide [44] and so we can now seek to identify preferred conformations and interactions from the mutagenesis data given in table 1, which indicates the most important residues for CGRP or AM binding.

**Table 1.** Data used to select loop conformations.

CLR residues	CGRP effect	AM effect
A273	√	√
R274	√	√
Y277	√	
Y278	√	
D280	√	√
C282	√	
W283	√	√
I284	√	√
S285	√	√
D287	√	
T288	√	√
L290	√	
L291	√	

The residue that is the equivalent to position 6 in CGRP is conserved as threonine in all members of the CGRP/CT/AM/amylin family of peptides and is essential for CGRP agonist activity [44,50]. Sequence analysis shows that the most likely candidates that are (i) conserved in CLR and CTR and (ii) able to donate or accept an H-bond are in ECL2 where D280, D287 and T288 are the best candidates. As D280 makes few interactions to T^6^ (see above) and D287 is not significant in AM binding (figure 1) and is of moderate importance in CGRP activation, it would seem that T288 is the most promising candidate.

**Figure 5.**
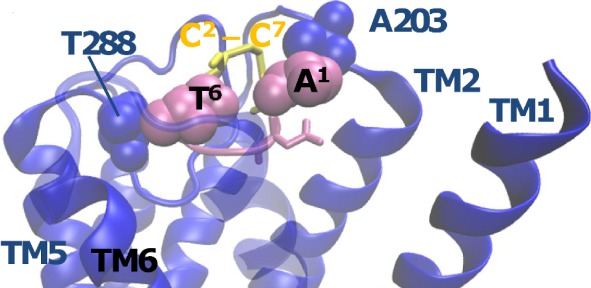
The highest scoring docked pose of CGRP_1–7_ (mauve). Resides A^1^ and T^6^ reside close to Ala203 and Thr288, respectively (blue). The CGRP disulfide bond is shown in yellow. The loop conformation shown here for ECL2 was a high scoring (i.e. favoured) conformation. TM7 is shown as transparent.

Figure 5 shows that the best-scoring docked conformation of CGRP_1–7_ satisfies the T^6^ criteria (by interacting with D280/D287/T288) in the presence of a sample ECL2 conformation (figure 4), and that D^3^ is not in a sterically crowded region (see also figure 4*c*). This indicates that the docked CGRP_1–7_ has provided a suitable template for modelling the full CGRP peptide and hence modelling the conformations of ECL2. In the docked conformation, A^1^ of CGRP is not only close to A203 of ECL1 of CGRP, but also able to accommodate N-terminal extensions. As discussed in the electronic supplementary material, this is consistent with mutagenesis and bioinformatic analysis of CGRP and AM binding.

#### 3.2.2. Filtering the ECL2 conformations

The number of interactions made by key ECL2 residues (table 1) to CGRP for each of the 100 loop conformations are displayed in figure 4*b* (*y*-axis). The ideal loop conformation should make an interaction between T^6^ of CGRP and D280, D287 or T288, have a large negative DOPE score, make a high number of key interactions and ideally have W283 in a vertical orientation (see below). The majority of conformations, denoted by a grey cross in figure 4*b*, do not make an appropriate interaction with T^6^ and are discarded. Several conformations make 6–9 interactions, including those to T^6^ via D280, D287 or T288. Because interactions to D280, D287 or T288 are observed in the top 15% of the most energetically preferred conformations and because the DOPE score is an empirical rather than a rigorously accurate score, it is not advisable to use energy (i.e. the DOPE score) as the sole criteria to identify the preferred binding mode, hence the importance of filtering the loop conformations using the mutagenesis data. Only one conformation (conformation 34, top left of figure 4*b*) records a direct interaction with R274^4.64^, for either CGRP or AM, but this interaction is to R^11^ of CGRP and the distance is long; closer inspection shows that D280 bridges between R274^4.64^ and R^11^ of CGRP. Several other conformations of R274^4.64^ act in this way, and could help to orientate a D280-R^11^ interaction. Thus, a direct interaction between CGRP and R274^4.64^ is probably an unrealistic selection criterion. R274^4.64^ is highly conserved as arginine or lysine across the class B GPCRs; mutation in the GLP-1 and secretin receptors also impairs function [6,7,51]. It is possible that the positively charged head group may also interact with the phospholipid bilayer [52].

**Figure 6.**
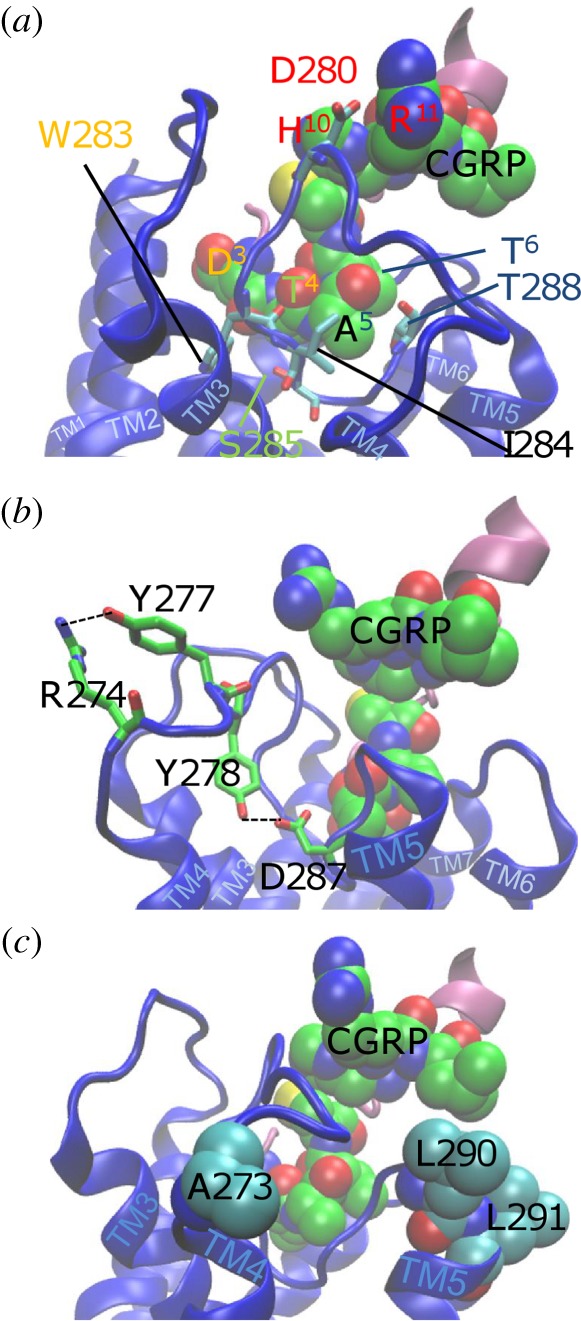
The interactions in the high scoring models for CGRP model. The interactions include D280 with H^10^ and R^11^, T288 with T^6^ (and A^5^), S285 with A^5^ and D287 with A^5^ (shown in (*b*); the ECL2 hydrophobic residue interactions include W283 with D^3^ and T^4^ as well as residues on TM2 and TM3, and I284 with A^5^. (*b*) Hydrogen bonds between significant ECL2 residues include R274 interacting with Y277 and D287 (close to A5) interacting with Y278, which seem to stabilize the ECL2 fold. (*c*) The remaining significant ECL2 residues, namely A273, L290 and L291; A273 and L291 do not interact with CGRP, L290 interacts with L^12^ in some ECL2 conformations while L291 points towards TM7, but could also affect the RAMP interaction; it is notable that most of these non-interacting residues plus Y277 and Y278 are not significant in the binding of AM and so it is likely that in CLR they are important in RAMP1-directed indirect effects on the binding. These structures have not been refined by molecular dynamics simulations for reasons discussed above and so the molecular information should not be overinterpreted.

Based on the data in figure 4*b* (and similarly for AM interactions with the CGRP receptor which altered pEC_50_), we selected six conformations for CGRP and seven for AM (see the electronic supplementary material, figure S4). Apart from one CLR/CGRP structure (conformation 34), these all have a similar conformation for ECL2. However, despite a similar ECL2 conformation, there are differences in the orientations of the W283 side chain, and only one high scoring CGRP and one high scoring AM conformation have W283 in a vertical orientation (a range of W283 interactions is shown in the electronic supplementary material, figures S4 and S5). The preferred CGRP structure (figure 6) satisfies 6/13 of the mutagenesis results given in table 1, whereas the AM model (see the electronic supplementary material, figure S6) satisfies 4/7.

#### 3.2.3. The orientation of W283

The loop generation alone does not help to address the orientation of W283 as it interacts with CGRP in most conformations of ECL2. However, the docking raises an interesting question with regard to the site-directed mutagenesis data on TM2 and TM3, as shown in the electronic supplementary material, figure S7. A number of residues on TM2 and TM3 (namely T191^2.57^, L195^2.61^, V198^2.64^, A199^2.65^ and H219^3.32^) show reduced cAMP production on mutation [19]. In our model, CGRP can interact with L195^2.61^, V198^2.64^, A199^2.65^ but not T191^2.57^ and H219^3.32^ because they lie too deeply within the helical bundle. If CGRP were positioned to interact with these residues, it would then most likely not satisfy the interactions of A^1^ and T^6^. The alanine-substitution effect at T191^2.57^ and H219^3.32^ may instead result from interactions with W283 of ECL2 as suggested by analysis of inactive CLR simulations and selected high scoring loop conformations (figure 6; see the electronic supplementary material, figures S5–S7). The hydrophobic patch of L195^2.61^, V198^2.64^ and A199^2.65^ on TM2 is important in CLR for CGRP-mediated activation of cAMP production [20] but while this region is more polar in some GPCRs, alternative hydrophobic regions reside nearby in other family B GPCRs where W283 could bind. Consequently, we prefer the vertical conformation of W283 as no other conformation satisfies the mutation data on T191^2.57^ and H219^3.32^.

#### 3.2.4. AM binding

Our models can also explain the mutagenesis data for AM-mediated activation of the same receptor. It is proposed that AM sits in a very similar orientation to CGRP in the presumed binding pocket (see the supplementary material) but accesses a slightly different subset of ECL conformations. There are no direct interactions with R274^4.64^, Y277 and Y278, but in the case of CGRP, the model suggests that there are also few, if any, direct interactions with these residues and instead they work primarily to stabilize productive conformations of ECL2. In the case of CGRP, we suggest that the consequence of this is to strengthen the interactions between the contact points between the peptide and ECL2 and so enhance potency. AM probably makes fewer contacts with ECL2; RAMP2 may be required to generate a full complement of interactions with this loop.

#### 3.2.5. Implications for receptor activation

The binding of an agonist has to stabilize an active conformation of the receptor, increasing the activity of effector proteins. For the CGRP receptor, the best characterized interaction is with Gs, leading to the production of cAMP and, based on homology with the active crystal structures of rhodopsin and the β_2_-AR and extensive mutagenesis, we produced a model of how this can take place [19]. Based on the current data, it is possible to make some broad points in regard to possible agonist activation mechanisms in CLR. A change in the C-terminal region of ECL2 could easily be propagated to TM5 and changes in this helix that are linked to movement of TM6 have been identified as one of the earliest steps leading to receptor activation in family A GPCRs [11,53]. It may be significant that T^6^ of CGRP, important for agonist activity in both CGRP and AM [44,50], is in close proximity to the C-terminal region of ECL2 where D287 or preferably T288 are its most likely interaction partners. Further support for an important role of TM5 in receptor activation comes from the disrupting effect of the double mutant of L220/L221A. A shift in TM5 in CLR could subsequently allow movement of TM6. In family B GPCRs, there is a conserved proline residue in TM6, which is likely to produce a kink [23] analogous to the situation found in family A GPCRs, so displacement of TM6 will lead to the opening of a G protein-binding pocket between it and TM3 [11].

The conserved N-terminal part of ECL2 [1] links ECL2 not only with TM4 but is also likely to influence ECL1 and hence TM2 and 3. While ECL1 is of only minor importance in the binding of CGRP, residues just below it in TM2 and H219^3.32^ of TM3 are of considerable significance for receptor activation [20]. Similar clusters are not obvious in the upper regions of the other TMs [19], although systematic mutagenesis is needed to test this. These residues at the tops of TM2 and 3 may be in a position to make contacts with residues at the base of ECL2, such as W283 and I284 as proposed in this study (see the electronic supplementary material, figure S7). Thus, CGRP has the potential to influence TM2 and 3 both directly and indirectly via ECL2. In family A GPCRs, TM3 is of particular importance for receptor activation partly owing to its angle of tilt across the TM bundle linking different parts of the bundle together. TM3 also constrains the C-terminal end of TM6 in at least some receptors via an ionic or polar lock [11,54]. In family B GPCRs, the equivalent of the ionic lock is probably a set of polar interactions involving residues at the C-terminal ends of TMs 2, 3 and 6 [19,22,55]; there are further interactions involving a polar network in the mid-regions of TMs 2, 3 and 7 [19,22,56]. Any interaction with TMs 2 and 3 is likely to play a key role in the activation of a family B GPCR.

The model developed for CGRP binding is therefore consistent with what is known about how CGRP activates its receptor, although it is speculative. It is also important to note that we have interpreted the effects of the mutations directly on the conformation of ECL2 itself. We cannot rule out that some of the effects may be on the TM bundle or extracellular domains of CLR or even of the RAMP. The data can be used to address these issues more fully once a crystal structure of the active CLR/RAMP1 complex becomes available.

## 4. Conclusion

We have evaluated the role of ECL2 in the binding of both CGRP and AM to the CGRP receptor and have interpreted the results by means of molecular modelling. The study indicates that ECL2 is particularly important for the interaction of CGRP with its receptor involving 13 ECL2 residues in the loop out of 24 residues studied. By contrast, only two residues in ECL1 and one in ECL3 influence the interaction of CGRP with its receptor [19,20]. Within ECL2, R274, W283, D280, D287 and T288 are of particular importance. We have suggested mechanisms where binding to this loop causes changes to the extracellular ends of TMs 2, 3 and 5, which in turn can be linked to movements of the cytoplasmic ends of TMs 3, 6 and 7, to allow G protein binding and activation.

While ECL2 appears to be involved in the binding of many peptide agonists to family B GPCRs, its precise role is probably receptor and ligand dependent. Models have been produced based on either mutagenesis or cross-linking data for the binding of secretin, GLP-1, VIP and PTH to their receptors [8,15,16,57,58]. While there are of course limitations to any modelling study, it is striking that there is no agreement as to the mode of binding. In the case of some models developed for GLP-1, secretin and VIP, their N-termini penetrate more deeply to interact with TM2, providing a more direct method of altering the conformation of both this helix and probably the adjacent TM3 during the process of receptor activation. While GLP-1 may not bind in the same way as CGRP (where the disulfide bond makes the N-terminus more bulky), a study of this receptor has provided the clearest evidence yet that ECL2 is an important determinant of signalling specificity [6,7]. Even in possession of crystal structures, the flexible nature of ECLs requires studies such as this to shed more light on how GPCRs respond to peptide agonists. Our approach used to model the interaction between either CGRP or AM and the receptor may be applicable to other GPCRs. The full set of structures is available from ftp.essex.ac.uk/pub/oyster/CLR_ECL2_2013/CLR_ECL2_structures.tar.gz (see also the electronic supplementary material); these structures compare favourably to the recent class B X-ray structures of glucagon and the corticotropin-releasing factor-1 receptors as described in the electronic supplementary material.
